# Supplementary material for: Common and rare genetic variants predisposing females to unexplained recurrent pregnancy loss
Source: Nat Commun. 2024 Jul 17;15:5744. doi: 10.1038/s41467-024-49993-5 (PMC11255296; doi:10.1038/s41467-024-49993-5)
Supplement: Supplementary file 1 — Supplementary Information [file 41467_2024_49993_MOESM1_ESM.pdf]

Supplementary Information for

Common and rare genetic variants predisposing females to unexplained recurrent pregnancy loss

K Sonehara and Y Yano et al.

Corresponding to Yukinori Okada (yuki-okada@m.u-tokyo.ac.jp) and
Mayumi Sugiura-Ogasawara (og.mym@med.nagoya-cu.ac.jp)

Table of Contents

Supplementary Figures ········· **3**

Supplementary Figure 1 | Quantile-quantile plot of the association *P*-values in the uRPL GWAS ········· 3

Supplementary Figure 2 | Stratified association analysis according to clinical features ········· 4

Supplementary Figure 3 | Number of detected copy-number variations per individual ········· 5

Supplementary Figure 4 | Expression overview of *CDH11* across tissues in the Human Protein Atlas ········· 6

Supplementary Figure 5 | Visualization of the principal component vectors of the GWAS participants ········· 7

Supplementary Data 1 and 2 are provided as separate .xlsx files.

Supplementary Figures

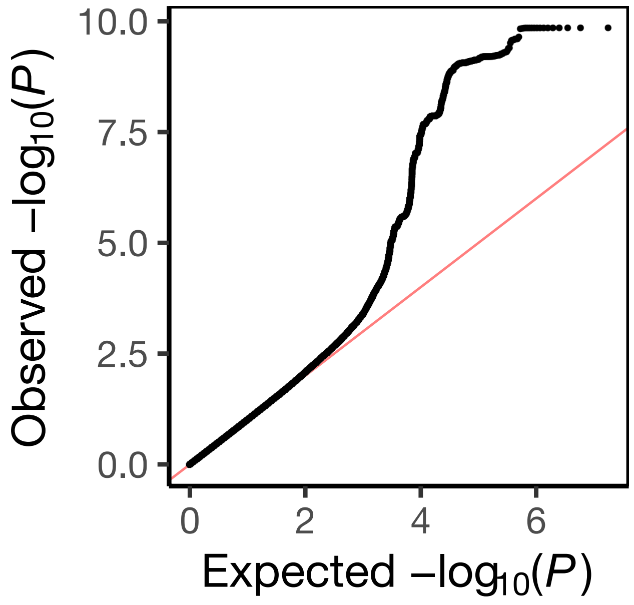

Supplementary Figure 1 | Quantile-quantile plot of the association *P*-values in the uRPL GWAS

The *x*-axis indicates the expected -$\log_{10}$(*P*) under the null hypothesis, and the *y*-axis indicates the observed -$\log_{10}$(*P*) in the GWAS. The red straight line indicates the identity line.

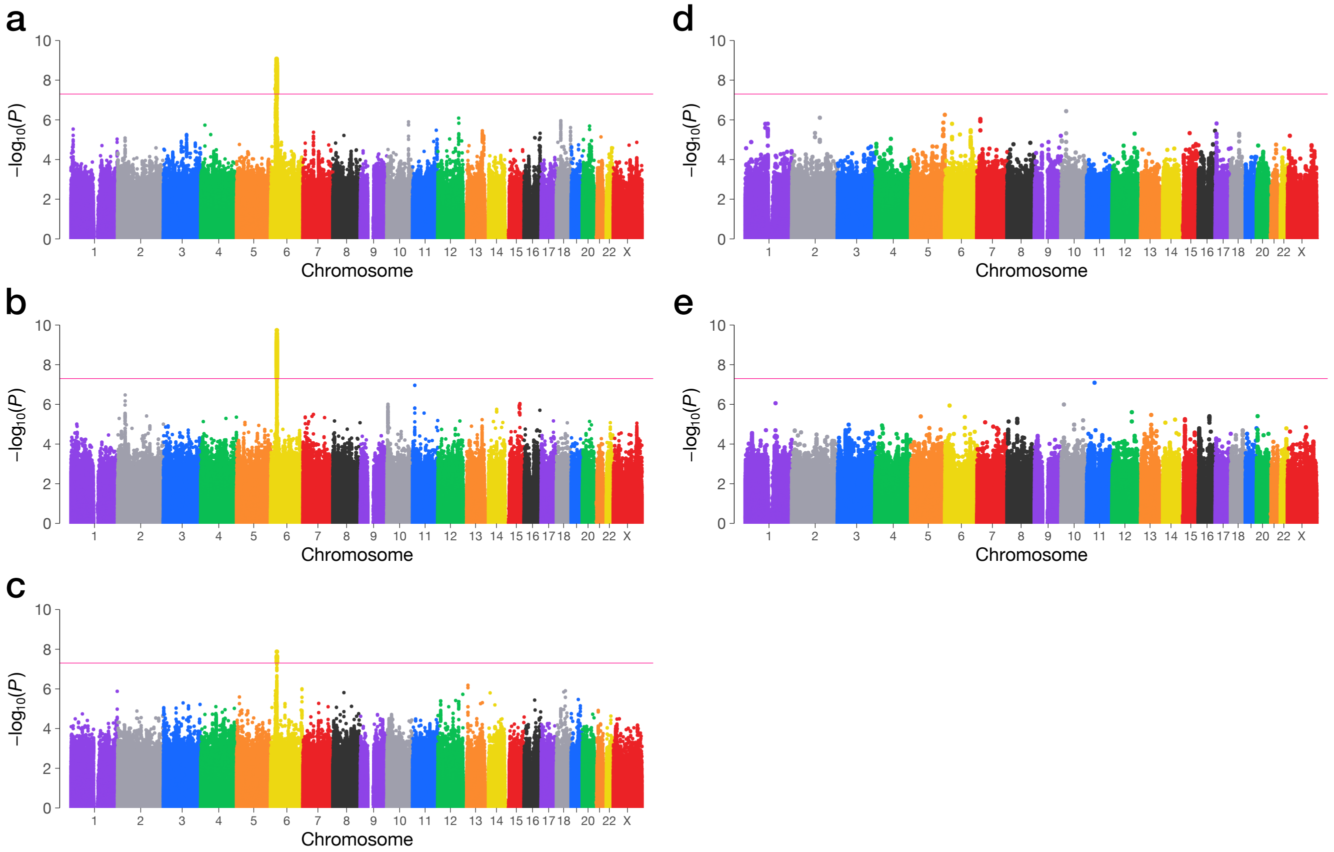

Supplementary Figure 2 | Stratified association analysis according to clinical features

Genome-wide associations of imputed genetic variants are shown. The pink horizontal line indicates the genome-wide significance threshold of $P = 5.0 \times 10^{-8}$. **a**, case group (A), uRPL cases with confirmed embryonic euploid or undetermined karyotype. **b**, case group (B), ANA(+) uRPL cases with confirmed embryonic euploid or undetermined karyotype. **c**, case group (C), autoantibody and hypothyroidism(-) uRPL cases with confirmed embryonic euploid or undetermined karyotype. **d**, case group (D), uRPL cases with confirmed embryonic euploidy. **e**, case group (E), uRPL cases with confirmed embryonic aneuploidy. *P*-values were computed using SAIGE. All statistical tests are two-sided and unadjusted for multiple comparisons.

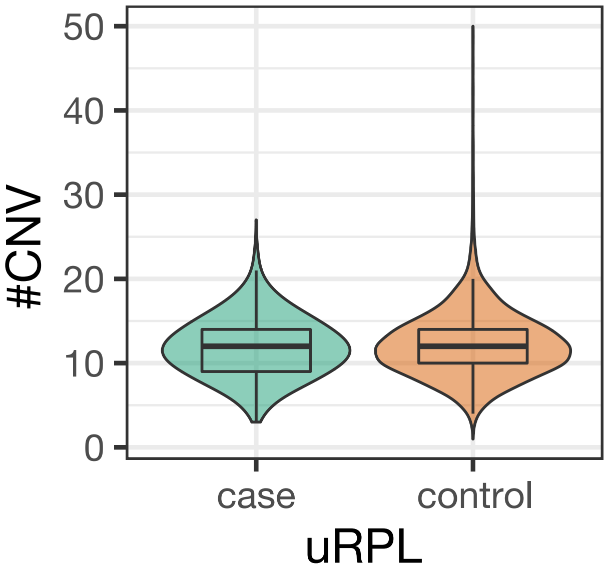

Supplementary Figure 3 | Number of detected copy-number variations per individual

Distributions of the number of detected copy-number variations per individual are shown separately for cases and controls. Boxplots represent the interquartile range (IQR), and the ends of whiskers represent the minimum and maximum values within 1.5 × IQR.

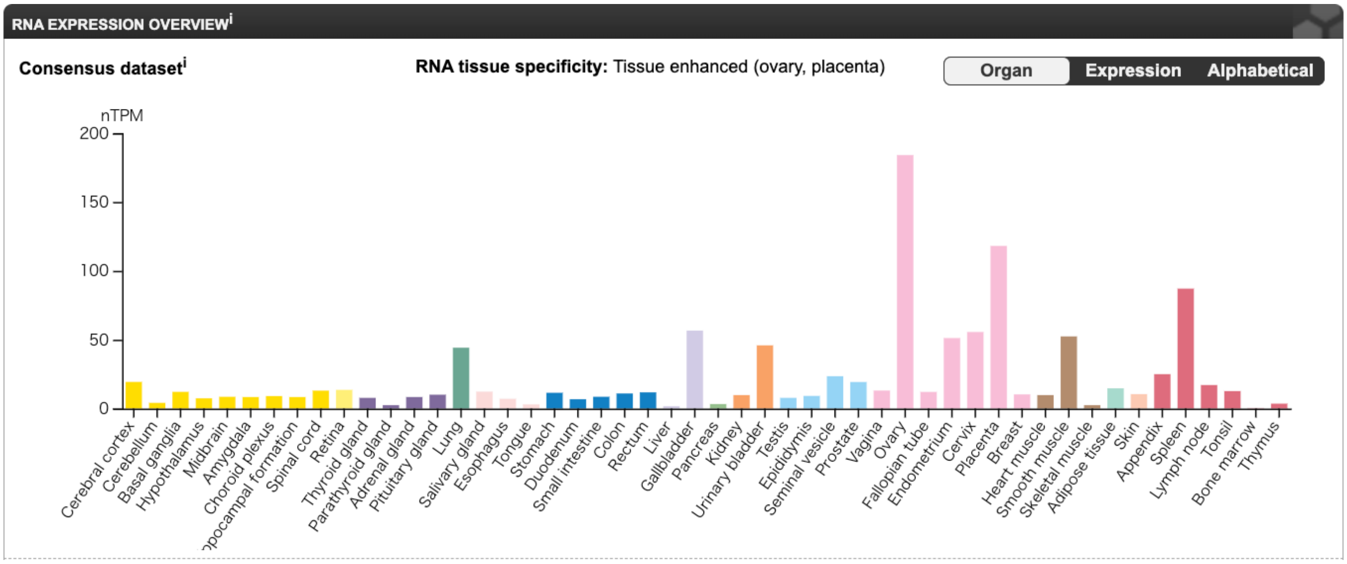

Supplementary Figure 4 | Expression overview of *CDH11* across tissues in the Human Protein Atlas

Bar charts of the *CDH11* expression levels are shown for the 50 tissues available in the consensus dataset provided by the human protein atlas normalization pipeline. The pink bar charts correspond to the tissue group annotated as "breast and female reproductive system". The image is available from the Human Protein Atlas released under a Creative Commons Attribution-Sharealike 3.0 license (https://v23.proteinatlas.org/ENSG00000140937-CDH11/tissue).

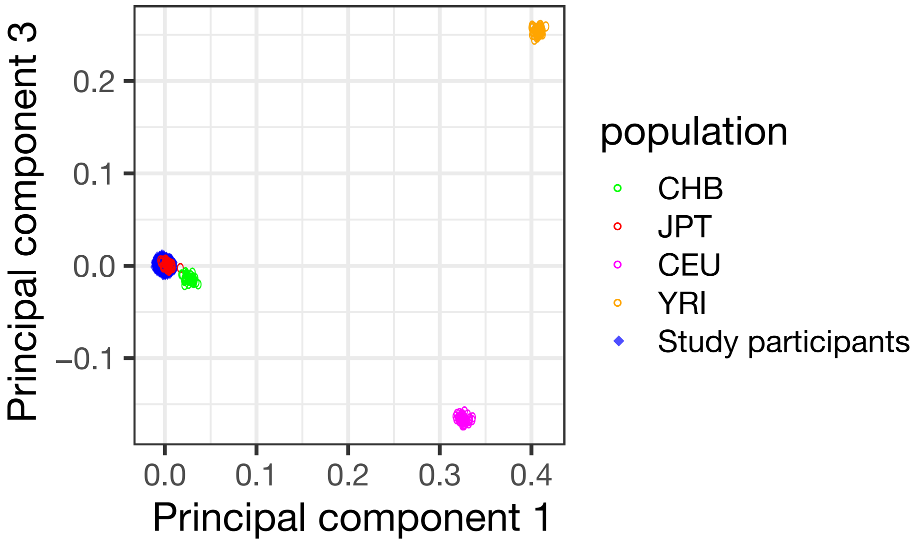

Supplementary Figure 5 | Visualization of the principal component vectors of the GWAS participants

The distributions of principal components of the genotypes of the study participants are indicated together with the individuals of the HapMap project. Each marker represents an individual. CHB, Han Chinese in Beijing, China; JPT, Japanese in Tokyo, Japan; CEU, Utah residents with Northern and Western European ancestry; YRI, Yoruba in Ibadan, Nigeria.
