# Supplementary material for: Common and rare genetic variants predisposing females to unexplained recurrent pregnancy loss
Source: Nat Commun. 2024 Jul 17;15:5744. doi: 10.1038/s41467-024-49993-5 (PMC11255296; doi:10.1038/s41467-024-49993-5)
Supplement: Supplementary file 3 — Description of Additional Supplementary Files [file 41467_2024_49993_MOESM3_ESM.pdf]

Description of Additional Supplementary Files

File Name: Supplementary Data 1

Description: Detailed association results of the HLA variants in the MHC region with uRPL risk

File Name: Supplementary Data 2

Description: Detailed association results of the pLoF CNVs with uRPL risk
