# Supplementary material for: Common and rare genetic variants predisposing females to unexplained recurrent pregnancy loss
Source: Nat Commun. 2024 Jul 17;15:5744. doi: 10.1038/s41467-024-49993-5 (PMC11255296; doi:10.1038/s41467-024-49993-5)
Supplement: Supplementary file 6 — Reporting Summary [file 41467_2024_49993_MOESM6_ESM.pdf]

nature portfolio

Corresponding author(s): Yukinori Okada, Mayumi Sugiura-Ogasawara

Last updated by author(s): May 30, 2024

Reporting Summary

Nature Portfolio wishes to improve the reproducibility of the work that we publish. This form provides structure for consistency and transparency in reporting. For further information on Nature Portfolio policies, see our Editorial Policies and the Editorial Policy Checklist.

Statistics

For all statistical analyses, confirm that the following items are present in the figure legend, table legend, main text, or Methods section.

n/a	Confirmed	
☐	☒	The exact sample size (*n*) for each experimental group/condition, given as a discrete number and unit of measurement
☐	☒	A statement on whether measurements were taken from distinct samples or whether the same sample was measured repeatedly
☐	☒	The statistical test(s) used AND whether they are one- or two-sided<br>*Only common tests should be described solely by name; describe more complex techniques in the Methods section.*
☐	☒	A description of all covariates tested
☐	☒	A description of any assumptions or corrections, such as tests of normality and adjustment for multiple comparisons
☐	☒	A full description of the statistical parameters including central tendency (e.g. means) or other basic estimates (e.g. regression coefficient) AND variation (e.g. standard deviation) or associated estimates of uncertainty (e.g. confidence intervals)
☐	☒	For null hypothesis testing, the test statistic (e.g. F, t, r) with confidence intervals, effect sizes, degrees of freedom and P value noted<br>*Give P values as exact values whenever suitable.*
☒	☐	For Bayesian analysis, information on the choice of priors and Markov chain Monte Carlo settings
☒	☐	For hierarchical and complex designs, identification of the appropriate level for tests and full reporting of outcomes
☒	☐	Estimates of effect sizes (e.g. Cohen's *d*, Pearson's *r*), indicating how they were calculated

Our web collection on statistics for biologists contains articles on many of the points above.

Software and code

Policy information about availability of computer code

Data collection	No software was used in data collection.
Data analysis	We used the following publicly available software for the data analysis: GenomeStudio 2.0.4, R version 3.6.3, PLINK 1.90b4.4 and 2.00a3LM, SHAPEIT4 version 4.2.1, Minimac4 version 1.0.1, DEEP*HLA, SAIGE version 0.44.6.1.26, LDSC version 1.0.1, and HI-CNV version 1.0

For manuscripts utilizing custom algorithms or software that are central to the research but not yet described in published literature, software must be made available to editors and reviewers. We strongly encourage code deposition in a community repository (e.g. GitHub). See the Nature Portfolio guidelines for submitting code & software for further information.

Data

Policy information about availability of data

All manuscripts must include a data availability statement. This statement should provide the following information, where applicable:

- Accession codes, unique identifiers, or web links for publicly available datasets
- A description of any restrictions on data availability
- For clinical datasets or third party data, please ensure that the statement adheres to our policy

GWAS genotype data of the BBJ are available at the National Bioscience Database Center (NBDC) Human Database (https://humandbs.dbcls.jp/en/) with the accession number of hum0311. The GWAS summary statistics will be available at the NBDC Human Database with the accession number of hum0197.

Research involving human participants, their data, or biological material

Policy information about studies with human participants or human data. See also policy information about sex, gender (identity/presentation), and sexual orientation and race, ethnicity and racism.

Reporting on sex and gender	We used the term sex as with other genomic studies. We collected clinical information, including sex, from medical records in Nagoya City University Hospital or BBJ. Due to the study design focusing on female pregnancy loss, all the analyses were performed in females.
Reporting on race, ethnicity, or other socially relevant groupings	The individuals included in the analysis were all Japanese.
Population characteristics	1,728 patients with unexplained recurrent pregnancy loss and 24,315 control individuals from BBJ were included in the study. All the study participants were female. The median age of the study participants at recruitment was 34 years old in the cases and 66 years old in the controls.
Recruitment	The patients with unexplained recurrent pregnancy loss were recruited from Nagoya City University Hospital between 2007 and 2022. The control individuals were registered in BBJ, a multi-institutional hospital-based biobank that collects DNA and medical records from individuals with at least one of 38 target diseases in Japan between 2013 and 2017.
Ethics oversight	This study was conducted with the approval of the Research Ethics Committee of Nagoya City University Graduate School of Medical Sciences, the University of Tokyo, and Osaka University.

Note that full information on the approval of the study protocol must also be provided in the manuscript.

Field-specific reporting

Please select the one below that is the best fit for your research. If you are not sure, read the appropriate sections before making your selection.

☒ Life sciences ☐ Behavioural & social sciences ☐ Ecological, evolutionary & environmental sciences

For a reference copy of the document with all sections, see nature.com/documents/nr-reporting-summary-flat.pdf

Life sciences study design

All studies must disclose on these points even when the disclosure is negative.

Sample size	This case-control study includes 1,728 patients with unexplained recurrent pregnancy loss and 24,315 control individuals. We did not perform sample size calculation but included the maximum number of individuals fulfilling the QC criteria.
Data exclusions	We excluded samples meeting any of the following criteria: genotyping call rate < 98% or of non-East Asian ancestry.
Replication	We performed sensitivity analyses and stratified analyses to accounting for potential biases introduced by the control selection, body mass index gap, and comorbidities in the cases, confirming the robustness of the GWAS finding.
Randomization	Not applicable due to case-control study design.
Blinding	Not applicable due to case-control study design.

Reporting for specific materials, systems and methods

We require information from authors about some types of materials, experimental systems and methods used in many studies. Here, indicate whether each material, system or method listed is relevant to your study. If you are not sure if a list item applies to your research, read the appropriate section before selecting a response.

Materials & experimental systems

n/a	Involved in the study	
☒	☐	Antibodies
☒	☐	Eukaryotic cell lines
☒	☐	Palaeontology and archaeology
☒	☐	Animals and other organisms
☒	☐	Clinical data
☒	☐	Dual use research of concern
☒	☐	Plants

Methods

n/a	Involved in the study	
☒	☐	ChIP-seq
☒	☐	Flow cytometry
☒	☐	MRI-based neuroimaging

Plants

Seed stocks	*Report on the source of all seed stocks or other plant material used. If applicable, state the seed stock centre and catalogue number. If plant specimens were collected from the field, describe the collection location, date and sampling procedures.*
Novel plant genotypes	*Describe the methods by which all novel plant genotypes were produced. This includes those generated by transgenic approaches, gene editing, chemical/radiation-based mutagenesis and hybridization. For transgenic lines, describe the transformation method, the number of independent lines analyzed and the generation upon which experiments were performed. For gene-edited lines, describe the editor used, the endogenous sequence targeted for editing, the targeting guide RNA sequence (if applicable) and how the editor was applied.*
Authentication	*Describe any authentication procedures for each seed stock used or novel genotype generated. Describe any experiments used to assess the effect of a mutation and, where applicable, how potential secondary effects (e.g. second site T-DNA insertions, mosiacism, off-target gene editing) were examined.*
